# WNT11/ROR2 signaling is associated with tumor invasion and poor survival in breast cancer

**DOI:** 10.1186/s13046-021-02187-z

**Published:** 2021-12-15

**Authors:** Kerstin Menck, Saskia Heinrichs, Darius Wlochowitz, Maren Sitte, Helen Noeding, Andreas Janshoff, Hannes Treiber, Torben Ruhwedel, Bawarjan Schatlo, Christian von der Brelie, Stefan Wiemann, Tobias Pukrop, Tim Beißbarth, Claudia Binder, Annalen Bleckmann

**Affiliations:** 1 Department of Medicine A, Hematology, Oncology, and Pneumology, University Hospital Münster, 48149 Münster, Germany; 2 West German Cancer Center, University Hospital Münster, 48149 Münster, Germany; 3 Department of Hematology/Medical Oncology, University Medical Center Göttingen, 37099 Göttingen, Germany; 4 Department of Medical Bioinformatics, University Medical Center Göttingen, 37099 Göttingen, Germany; 5 Institute for Physical Chemistry, Georg August University Göttingen, 37075 Göttingen, Germany; 6 Department of Neurogenetics, Max Planck Institute of Experimental Medicine, 37075 Göttingen, Germany; 7 Department of Neurosurgery, University Medical Center Göttingen, 37099 Göttingen, Germany; 8 Division of Molecular Genome Analysis, German Cancer Research Center, 69120 Heidelberg, Germany; 9 Department of Internal Medicine III, Hematology and Medical Oncology, University Hospital Regensburg, 93053 Regensburg, Germany

**Keywords:** Breast cancer, Metastasis, ROR2, WNT11, BRCAness, Network analysis

## Abstract

**Background:**

Breast cancer has been associated with activation of the WNT signaling pathway, although no driver mutations in WNT genes have been found yet. Instead, a high expression of the alternative WNT receptor ROR2 was observed, in particular in breast cancer brain metastases. However, its respective ligand and downstream signaling in this context remained unknown.

**Methods:**

We modulated the expression of ROR2 in human breast cancer cells and characterized their gene and protein expression by RNA-Seq, qRT-PCR, immunoblots and reverse phase protein array (RPPA) combined with network analyses to understand the molecular basis of ROR2 signaling in breast cancer. Using co-immunoprecipitations, we verified the interaction of ROR2 with the identified ligand, WNT11. The functional consequences of WNT11/ROR2 signaling for tumor cell aggressiveness were assessed by microscopy, impedance sensing as well as viability and invasion assays. To evaluate the translational significance of our findings, we performed gene set enrichment, expression and survival analyses on human breast cancer brain metastases.

**Results:**

We found ROR2 to be highly expressed in aggressive breast tumors and associated with worse metastasis-free survival. ROR2 overexpression induced a BRCAness-like phenotype in a cell-context specific manner and rendered cells resistant to PARP inhibition. High levels of ROR2 were furthermore associated with defects in cell morphology and cell-cell-contacts leading to increased tumor invasiveness. On a molecular level, ROR2 overexpression upregulated several non-canonical WNT ligands, in particular WNT11. Co-immunoprecipitation confirmed that WNT11 indeed interacts with the cysteine-rich domain of ROR2 and triggers its invasion-promoting signaling via RHO/ROCK. Knockdown of WNT11 reversed the pro-invasive phenotype and the cellular changes in ROR2-overexpressing cells.

**Conclusions:**

Taken together, our study revealed a novel auto-stimulatory loop in which ROR2 triggers the expression of its own ligand, WNT11, resulting in enhanced tumor invasion associated with breast cancer metastasis.

**Supplementary Information:**

The online version contains supplementary material available at 10.1186/s13046-021-02187-z.

## Background

Breast cancer is the most common cancer in women with more than 2 million new cases diagnosed in 2018 [1]. Patients frequently develop metastases in the course of their disease which limit survival due to the lack of a curative treatment. One signaling pathway that is frequently involved in cancer initiation and progression is the WNT pathway. In mammals it comprises 19 secreted WNT ligands that can interact with ten different Frizzled (FZD) receptors and various co-receptors [2]. WNT ligands activate different intracellular signaling cascades depending on the specific combination of locally available ligands, receptors and co-receptors.

Binding of a canonical WNT ligand (e.g. WNT3A) to a FZD receptor and LRP5/6 co-receptor activates β-catenin-dependent, canonical signaling that results in the expression of WNT-responsive target genes [2]. Other WNT ligands such as WNT5A/B, or WNT11 can bind FZDs and alternative co-receptors (e.g. ROR1/2, RYK, PTK7) and trigger a multitude of β-catenin-independent, non-canonical WNT signaling cascades, the best-studied ones being WNT/Calcium (Ca^2+^) and WNT/planar cell polarity (PCP) signaling. Activation of the WNT/Ca^2+^ pathway is characterized by an increase in intracellular calcium levels that triggers the activation of PKC, NFκB and CREB [3]. In contrast, in WNT/PCP signaling binding of a WNT ligand induces the recruitment of a DVL/RHO/DAAM1 complex that is required for subsequent activation of the RHO/ROCK pathway [4]. In parallel, DVL can activate the small GTPase RAC and downstream JNK signaling [5]. Non-canonical WNT signal transduction thus mostly results in changes in the cytoskeleton, cell motility and morphology.

While aberrant WNT signaling is a hallmark of colorectal cancer, its role in breast cancer is less clear. No driver mutations in typical WNT genes have been detected so far. Nonetheless, several studies point to hyperactive and dysbalanced WNT signaling [6, 7]. Especially in basal-like breast cancer, the most unfavorable clinical subtype with early metastasis formation, active non-canonical WNT signaling has been identified and linked to the aggressive behavior of these breast cancer cells. The highly motile and invasive phenotype of the cancer cells has been mainly attributed to the high expression of WNT5A/B and receptor tyrosine kinase-like orphan receptors 1 and 2 (ROR1/2) [8–11]. In contrast to ROR1, ROR2 was not only found to be highly expressed in basal-like but in 87% of all breast cancers, and high levels were associated with shorter overall survival [12, 13]. A significant role of ROR2 in tumor development has already been confirmed in vivo in a basal-like TP53-null mouse model of breast cancer where knockdown of ROR2 significantly impaired tumor growth [14]. Apart from the primary tumor tissue, high levels of ROR2 were detectable in lymph node and brain metastases [8, 13], thus suggesting its involvement in tumor progression and metastasis.

ROR2 harbors a cysteine-rich domain (CRD) in its extracellular part that resembles the WNT protein binding domain of FZD receptors. Indeed, ROR2 has been shown to interact with WNT5A and WNT3A, however, the latter failed to activate ROR2-induced signaling [15]. Considering the ambiguous role of WNT5A which has been described to act both as an oncogene as well as a tumor suppressor in breast cancer, it is still unclear whether other WNT ligands exist that can activate ROR2 signaling.

In this study we addressed this question and demonstrated for the first time that human WNT11 acts as a ligand for ROR2. WNT11 binds to the CRD of ROR2 and mediates WNT/PCP signaling via the RHO/ROCK pathway that confers an aggressive phenotype to breast cancer cells. ROR2 and WNT11 are both highly expressed in human brain metastases and linked with short patient survival.

## Methods

### Cell lines, transfections and viability assays

MCF-7, T-47D, MDA-MB-231 and SK-BR-3 cells (DSMZ, ATCC) were cultured in RPMI-1640, BT-474 cells in DMEM/F12, all supplemented with 10% heat-inactivated (56 °C, 30 min) fetal calf serum (FCS). For gene knockdown, cells were transfected with 10 nM siRNA (santa cruz) using RNAimax (Invitrogen). Cells were used 24 h post transfection for functional studies and 72 h post transfection for expression analysis. For gene overexpression, cells were transfected with Fugene HD (Promega) and stable clones selected by geneticin or zeocin selection (750 μg/ml or 100 μg/ml, respectively). Cell viability upon treatment with olaparib (Selleck chemicals) for 96 h was measured by MTT assay using standard protocols.

### Vectors

The vector for V5-tagged active WNT11 was a gift from Xi He (Addgene plasmid #43824; http://n2t.net/addgene:43824; RRID:Addgene_43,824) [16]. The pcDNA3.1/Zeo(+) empty vector was obtained from Invitrogen. The pROR2 vector was kindly provided by Alexandra Schambony. N-terminal ROR2 deletion constructs were generated by PCR-based cloning. They consisted of amino acids 146–943 for ROR2-Δ, 304–943 for ROR2-ΔΔ and 395–943 for ROR2-ΔΔΔ. C-terminal truncation was achieved by creating premature stop codons at amino acid position 467 (ΔPRD) and 783 (ΔPRDΔTKD) using site-directed mutagenesis. Successful cloning was confirmed by sequencing.

### RNA-Seq data retrieval from TCGA

Breast invasive carcinoma TCGA PanCancer data [17–19] from 1084 patients were retrieved from cBioPortal (https://www.cbioportal.org/study/summary?id=brca_tcga_pan_can_atlas_2018). Clinical annotations were pre-filtered for patients with PFS > 0 months. Raw count data corresponding to the samples were imported using the TCGAbiolinks R package (v.2.22.1) [20] for the TCGA RNA-Seq dataset (“HTSeq-counts” files). For downstream analysis, gene-level counts were converted to log2-transformed normalized counts as transcripts per million.

### Patient samples

Samples of brain metastases were collected from patients previously diagnosed with primary breast cancer during neurosurgical removal. Expression of *ROR1*, *ROR2*, *PTK7* and *RYK* in normal and cancerous breast was analyzed on RNA-Seq data from matched samples of normal and invasive breast carcinoma tissue using the TNMplot database (https://www.tnmplot.com/) [21]. Microarray data of primary breast cancer patients were compiled as previously described [10].

For RNA-Seq analysis of breast cancer brain metastases total RNA was isolated with the Trizol reagent from fresh frozen tissue and RNA integrity was checked with the Bioanalyzer 2100 (Agilent Technologies). For cDNA library preparation the TruSeq Stranded Total RNA sample preparation kit (Illumina) was used, and accurate quantification was performed with the QuantiFluor dsDNA system (Promega). The size range of the generated cDNA libraries was measured with the DNA 1000 chip on the Bioanalyzer 2100 (280 bp). Amplification and sequencing of cDNA libraries were performed using the cBot and HiSeq 2000 (Illumina, SR, 1 × 51 bp, 8–9 gigabases, > 40 mio reads per sample). Sequence images were transformed with Illumina software BaseCaller to bcl-files, which were demultiplexed to fastq-files with CASAVA (v1.8.2). Quality check was done via FastQC (v0.10.1, Babraham Bioinformatics).

Sequence reads were aligned with the reference genome GRCh37 using the STAR RNA-Seq alignment tool [22], while incorporating database information from Ensembl (v37.73) during the reference indexing step. Gene-level abundances were estimated using the RNA-seq by expectation-maximization (RSEM) algorithm [23]. RSEM estimated counts of 31 brain metastases samples were converted to log2-transformed normalized counts as transcripts per million.

### WNT pathway enrichment analysis

Rank-based enrichment testing was performed for three different WNT pathway signatures using the Wilcoxon rank-sum test, thereby obtaining *P-*values across patient samples per pathway [10]. Transformed -log10(*P-*values) were then subjected to complete-linkage hierarchical clustering based on Pearson correlation as the distance measure. Patient groups in terms of high and low pathway enrichment were obtained using the *cutree* function from the *dendextend* R package [24].

### Survival analysis

To conduct Kaplan–Meier survival analysis of gene expression levels, high/low groups were defined based on gene expression levels for single genes or averaged gene expression levels for gene signatures by applying an optimal cutoff value that was computed using the *surv_cutpoint* function from the *survminer* R package (v0.4.8, https://CRAN.R-project.org/package=survminer). Kaplan–Meier survival analysis were performed to assess different survival rates between the groups using the Log-Rank test. Significance *p* values and hazard ratios (HR) were obtained using the *survival* R package.

### RNA-Seq data of cell lines

RNA-Seq of MCF-7 pROR2 cells is described in [10]. For SK-BR-3 pROR2 cells, RNA was isolated using the High Pure RNA isolation kit (Roche). Following mRNA library preparation, subsequent sequencing was performed on the NextSeq 2000 system (Illumina, 1 × 72 cycles, > 25 mio single reads per sample). Quality check was done via FastQC (v0.11.8, Babraham Bioinformatics). Sequence reads were aligned with the reference genome GRCh38 using the HISAT2 (v2.1.0) alignment tool [25], while incorporating database information from Ensembl (v38.87) during the reference indexing step. Gene-level abundances were estimated using featureCounts (v1.6.3) [26]. The gene-level count matrix was first filtered and genes with count per million (CPM) > 1 in more than half of the samples were kept using the *edgeR* R package [21]. The count matrix was then normalized using the “Trimmed Mean of M-values” method from the *edgeR* R package and log2-transformed normalized pseudo counts were considered for downstream analysis.

### PAM50 molecular subtype classification

Gene-level count values were first averaged over triplicates per condition for each gene in the matrix and probability percentage values were estimated for each condition to belong to one of the five molecular subtypes using the molecular.subtyping function from the *genefu* R Package.

### BRCAness-like gene signature scoring

To quantify the relevance of a BRCAness-like phenotype, the unsupervised gene set enrichment method called “Gene Set Variation Analysis” (GSVA) [27] was applied to the gene-level count matrix for three different BRCAness-associated gene sets [28–30]. GSVA enrichment scores per sample were computed using the *GSVA* R package with default settings. Gene expression levels were fitted to the recently described network of non-canonical WNT signaling [31] and network visualization was done with R package *igraph*.

### Identification of master regulators in networks

We pursued an approach called “upstream analysis” which aims to identify master regulators in non-canonical ROR2/WNT11 signaling pathways in MCF-7 breast cancer cells that can be used to find suitable points of intervention for cancer therapy. The upstream analysis strategy comprises three steps: (1) identification of differentially expressed genes (DEGs) based on pairwise comparisons, (2) state-of-the-art promoter analysis to identify relevant transcription factors which are likely to regulate the input DEGs and (3) search for upstream master regulators which are at the very top of the regulatory hierarchy in signal transduction pathways [32]. The main algorithm of the upstream analysis has been described earlier in [33, 34]. In the first step, gene-level counts of samples were processed using the R package *DESeq2* [35] to carry out differential expression analysis.

In more detail, DEGs between two conditions (each with triplicate) were identified using the DESeq2 results function with the explicit parameters: alpha = 0.05, lfcThreshold = log2(1.5) and altHypothesis = “greaterAbs”. Log fold changes were shrunk using the R package *apeglm* [36] and genes with adjusted *P*-values (Benjamini-Hochberg method) less than 0.05 were considered to be DEGs. In the same step, we also compiled matching sets of non-DEGs for each pairwise comparison, that is, genes whose expression level did not change between tested conditions. The sets of non-DEGs are required for promoter analysis as background sets in the second step of the upstream analysis. For this purpose, we used the *DEseq* function with argument betaPrior = FALSE and the results function with the explicit parameters: alpha = 0.05, lfcThreshold = log2(1.5) and altHypothesis = “lessAbs” and genes with adjusted *P*-values less than 0.05 were considered to be non-DEGs.

The upstream analysis was concluded using the ready-to-use workflow called “Upstream analysis (TRANSFAC(R) and TRANSPATH(R))” of the geneXplain platform [32] web edition 6.2 (https://genexplain.gwdg.de/bioumlweb/) which incorporates the database TRANSFAC(R) [37] on transcription factors and their DNA binding sites as well as the pathway database TRANSPATH(R) [38]. In our analysis we have run this workflow using our matched lists of DEGs and non-DEGs per comparison as the Inputs for “Yes gene set” (target) and “No gene set” (background), respectively. We also restricted the search for potential transcription factor binding sites for the promoter analysis to the region of − 500 bp upstream (start of promoter) and 100 bp downstream (end of promoter) relative to the transcription start site and left all other parameters at their defaults including an FDR cutoff of 0.05 to retrieve significant master regulators.

### Reverse phase protein array (RPPA)

MCF-7 pcDNA/pROR2 cells were transfected with control (#sc-37007) or WNT11 siRNA (#sc-41120, both santa cruz) as described above. At 48 h post transfection the cells were lysed in M-PER buffer on ice and subjected to RPPA analysis. Briefly, lysates were mixed with 4x printing buffer (125 mM Tris pH 6.8, 10 mM DTT, 4% SDS, 10% glycerol), boiled for 5 min at 95 °C and printed in triplicates onto nitrocellulose-coated glass slides (Oncyte Avid, Grace Bio-Labs). For blocking, the slides were incubated for 2 h at room temperature in fluorescent western blot blocking buffer (ROCKville) mixed 1:1 with 5 mM NaF, 1 mM Na_3_VO_4_ in TBS pH 7.6 followed by incubation with the primary antibodies (Table S1) at 4 °C overnight. Slides incubated without primary antibody served as blank controls. Signals were detected using Alexa Fluor 680 F(ab’)2 fragments of goat anti-mouse or anti-rabbit IgG on an Odyssey infrared imaging system (LI-COR Biosciences). For normalization, separate slides were stained with Fast Green FCF for total protein quantification. Pre-processing, quality assessment and normalization of RPPA data were performed with the *RPPanalyzer* R package [39]. Differential proteins between the different conditions were identified by fitting linear models and significance calculated using empirical Bayes moderated t-statistics as implemented in the *limma* R package (v3.34.9) [40].

### Microscopy

For atomic force microscopy (AFM) cells were fixed with 4% PFA in PBS and cell surface scans recorded using an MFP-3D atomic force microscope (Asylum Research), equipped with a silicon nitride cantilever (MLCT, *k* = 10 mN·m^− 1^, Bruker AFM Probes). Images were recorded in contact mode in PBS at room temperature with a scan rate of 0.3 Hz. Image processing was performed using Gwyddion (http://gwyddion.net/) [41]. For ZO-1 and ROR2 immunofluorescence, samples were stained using an AlexaFluor488-labeled ZO-1 (#339188, Invitrogen) or ROR2 (#FAB20641G, R&D) antibody. Fluorescence was imaged on a confocal laser scanning microscope (Olympus, FV 1200 and Zeiss LSM800). Images were edited in ImageJ. For electron microscopy, the cells were fixed according to [42] for 24 h, then embedded in Epon post fixation with 1% OsO_4_, 1.5% uranyl acetate, 1.5% tungstophosphoric acid followed by dehydration with ethanol. A diamond knife was used to prepare ultra-thin sections (50 nm). Micrographs were obtained using a Zeiss EM 912 electron microscope.

### Electric cell-substrate impedance sensing (ECIS)

ECIS experiments were carried out on a homebuilt setup as described previously [43]. 3·10^5^ cells were incubated for 96 h and impedance was measured at 20 logarithmically spaced frequencies between 10 and 100,000 Hz over time. A cell-electrode model was fitted to the frequency-dependent complex impedance spectra from the time point the electrode was covered with a cell layer. The model parameter α, which is inversely proportional to the square root of the cell-substrate distance, was obtained as described previously [44]. Additionally, 1·10^4^ cells per well were seeded onto E-Plates 16 and analyzed in the xCELLigence RTCA DP system (Roche) for 48 h in quadruplicates.

### Western blotting and co-immunoprecipitation

To analyze protein expression, cells were lysed in RIPA buffer (50 mM Tris, 150 mM NaCl, 0.1% SDS, 0.5% sodium deoxycholate, 1% Triton X-100, pH 7.2) supplemented with protease (Sigma) and phosphatase (Roche) inhibitors. Up to 75 μg of protein were separated by SDS-PAGE, blotted onto nitrocellulose and incubated overnight at 4 °C with primary antibodies (Table S2). Membranes were incubated with HRP-coupled secondary antibodies (santa cruz, CST) for 1 h at RT and chemiluminescence detected with ECL Prime (GE Healthcare) at the LAS-1000 (Fujifilm), Amersham Imager 600 (GE Healthcare) or ChemoStar Touch Imager (Intas). Image J software was used for densitometric quantification. For co-immunoprecipitation, cells were transfected with V5-tagged WNT11 and 24 h post transfection proteins were crosslinked for 30 min with 1 mM DSS (Thermo Fisher) in 1 ml PBS + 1 mM MgCl_2_. Cells were lyzed in 50 mM Tris/HCl pH 8.0, 150 mM NaCl, 1% NP-40 + protease inhibitors. Five hundred micrograms protein were incubated with 1 μg normal rabbit IgG (#2729) or V5 antibody (#13202, both CST) for 16 h at 4 °C. Antibody-protein complexes were incubated for 2 h at 4 °C with protein A/G agarose beads (#sc-2003, santa cruz) and spun down at 1700 g for 1 min. Signals were visualized by western blot as described above using the confirmation-specific mouse anti-rabbit IgG (#3678, CST) for V5 detection.

### Gene expression analysis

Total RNA was extracted with the High Pure RNA isolation kit (Roche) and 1 μg reversely transcribed into cDNA (iScript cDNA synthesis kit, Bio-Rad). Gene expression was assessed from 10 ng cDNA at the ABI 7900 HT system using SYBR green detection and the SDS (v2.4) software (Applied Biosystems). For normalization the two housekeeping genes *HPRT1* and *GNB2L1* were used. Primer sequences are given in Table S3.

### Flow cytometry

Cells were stained for 20 min with a ROR2 Alexa Fluor 488-conjugated antibody (#FAB20641G) or the respective isotype control (#IC003G, both R&D), measured on an Attune NxT flow cytometer (ThermoFisher) and analyzed with FlowJo (v10.6.1).

### Cell invasion and migration

Cell invasion was measured in a modified Boyden chamber [9] and related to the unstimulated control. Cells were pre-incubated for 2 h with the indicated inhibitors.

### RHOA activity assay

MCF-7 and BT-474 cells were incubated in culture medium with + 1% FCS overnight. The next day, samples were stimulated with culture medium + 10% FCS for 30 min. Lysate preparation and measurement of RHOA-GTP levels were carried out with the RHOA G-LISA activation assay kit (#BK124, Cytoskeleton) according to the manufacturer’s instructions.

### Statistics

All experiments were carried out at least 3 times unless stated otherwise. Results are displayed as means ± standard deviation (SD). Statistical analyses were carried out with GraphPad Prism (v8.4.2) using a two-sided paired/unpaired *t*-test unless stated otherwise. *p* < 0.05 was considered statistically significant. All figures in the current study were either created with GraphPad Prism or generated in R (v3.6.2). The *ggsurvplot* function from the *survminer* R package was used to generate Kaplan–Meier survival plots. The functions *heatmap.2* and *pheatmap* from the R packages *gplots* (v3.1.0, https://CRAN.R-project.org/package=gplots) and *pheatmap* (v1.0.12, http://CRAN.Rproject.org/package=pheatmap) were used for heatmap visualizations.

## Results

### High ROR2 expression is associated with early metastasis in breast cancer patients

**Fig. 1:**
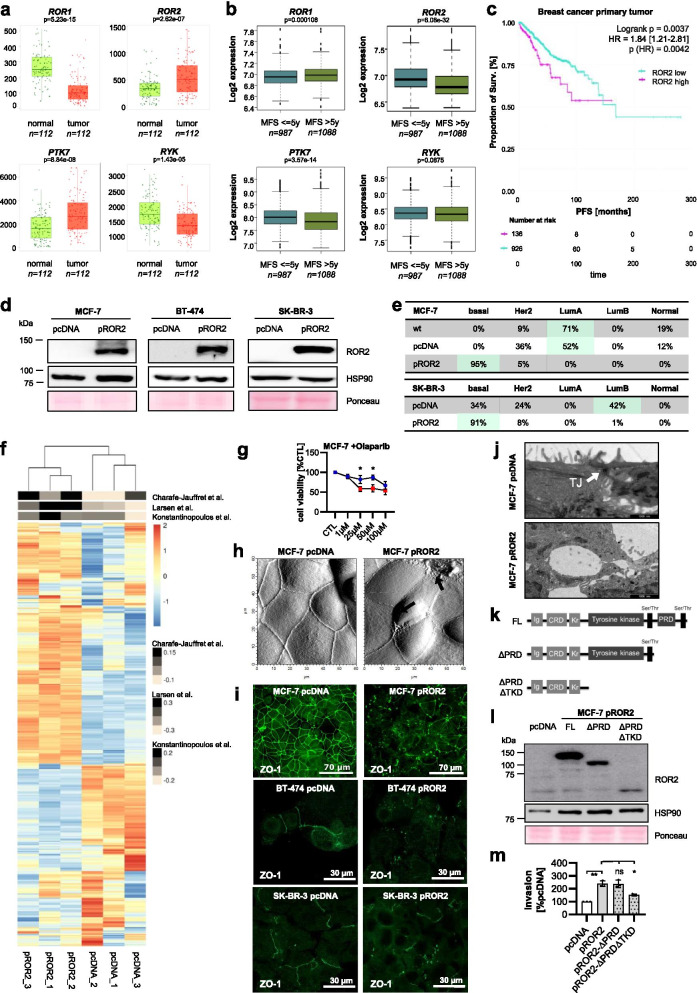
Expression of ROR2 induces a highly aggressive phenotype in breast cancer cells. **a** RNA-Seq: Gene expression of the four non-canonical WNT co-receptors *ROR1, ROR2, PTK7* and *RYK* in normal breast (green) and matched breast cancer tissue (red) from the TNMplot database. Significance was calculated with a paired Wilcoxon statistical test. **b** Microarray gene expression data from 2075 breast cancer primary tumors were correlated with either poor (≤ 5 years) or better (> 5 years) metastasis-free survival (MFS). Significance was calculated with a t-test. Boxes represent the 25-75th percentiles with the line at the median. Outliers are marked as small dots. **c** Progression-free survival (PFS) of 1067 breast cancer patients from TCGA stratified by ROR2 mRNA expression. **d** MCF-7, BT-474 and SK-BR-3 human breast cancer cells were transfected either with an empty vector (pcDNA) or with a hROR2 overexpression plasmid (pROR2). Transfection efficiency was confirmed by western blot. **e** Determination of the molecular breast cancer subtype based on RNA-Seq data from the indicated cell lines using the PAM50 gene signature. **f** RNA-Seq of MCF-7 cells: GSVA analysis for three independently published BRCAness gene expression signatures. **g** MTT assay: Cells were treated for 96 h with the indicated concentrations of olaparib (mean ± SD, *n* = 3, *p < 0.05). **h** Atomic force microscopy (AFM) of MCF-7 pcDNA and pROR2 cells. **i** Immunofluorescence staining of the tight junction marker ZO-1. **j** Electron microscopy of a representative tight junction (TJ) in MCF-7 pcDNA cells. An example for a defective cell-cell-junction is shown for pROR2 cells (scale bar: 1 μm). **k** Schematic overview of the pROR2 C-terminal deletion constructs. **l**+**m** C-terminal deletion constructs were overexpressed in MCF-7. Expression was confirmed by western blot (**l**) and the invasion rate measured in Boyden chambers (**m**) (mean ± SD, *n* = 3, *p < 0.001, **p < 0.0001, ns = not significant). Significance was calculated with a one-way ANOVA with Dunnett’s multiple comparison test

Based on the observations of active non-canonical WNT signaling in breast cancer tissue, we compared the expression levels of the known four non-canonical WNT co-receptors *ROR1*, *ROR2*, *PTK7* and *RYK* in normal and cancerous breast tissue using the TNMplot database (Fig. 1a) [21]. While *ROR2* as well as *PTK7* were expressed at significantly higher levels in cancerous tissue, *ROR1* and *RYK* expression was downregulated. We then investigated whether the expression levels of the receptors were correlated with the clinical prognosis using a compendium dataset, which comprises gene expression data of primary breast cancers from ten public datasets with a total of 2075 patients [10]. Indeed, the expression levels of *ROR2*, *PTK7* and *RYK* were negatively associated with metastasis-free survival (MFS) of the patients, although the effect was not statistically significant for the latter (Fig. 1b). In contrast, patients with a poor MFS displayed slightly lower ROR1 levels. These observations suggested that *PTK7* and *ROR2* contribute to an aggressive cancer cell phenotype that promotes early metastasis formation. Since the strongest effect on survival was seen for *ROR2*, we focused our further analyses on this receptor. In line with our hypothesis, analysis of the progression-free survival of 1067 breast cancer patients from the TCGA database confirmed the prognostic relevance of ROR2 and its association with aggressive disease (Fig. 1c).

### ROR2 expression induces an aggressive cancer cell phenotype linked to BRCAness

Next, we aimed to unravel the molecular events that underlie the unfavorable prognostic role of *ROR2*. Since ROR1 and ROR2 are known to interact and compensate for each other, we choose to limit potential cross-reactivity by stably overexpressing ROR2 in the ROR-negative [9] human breast cancer cell lines MCF-7, SK-BR-3 and BT-474 (Fig. 1d). Characterization of the ROR2-overexpressing (pROR2) MCF-7 and SK-BR-3 cells by RNA-Sequencing (RNA-Seq) revealed that overexpression of ROR2 changed the molecular characteristics of the cell lines from the rather benign luminal A and luminal B to the highly-aggressive basal-like subtype as calculated from the PAM50 gene signature (Fig. 1e). This is in correspondence with their increased invasive and migratory potential as well as with observations made in mouse models [9, 12, 14]. Since some triple-negative breast cancers share the high-grade genomic instability observed in BRCA1/2-mutant cancers [45], the so-called BRCAness, we were interested in whether ROR2 could be linked to these changes. Indeed, the gene expression profile of the ROR2-overexpressing MCF-7 cells showed a higher enrichment for three independently published BRCAness signatures [28–30] (Fig. 1f). Moreover, the cells showed increased susceptibility to treatment with the poly (ADP-ribose) polymerase (PARP) inhibitor olaparib (Fig. 1g). However, the induction of a BRCAness-like phenotype was restricted to MCF-7 cells, as the ROR2-overexpressing SK-BR-3 did not show an enrichment of BRCAness signatures or sensitivity toward PARP inhibition (Fig. S1). Since not all basal-like breast cancers are characterized by BRCAness, the two model cell lines MCF-7 and SK-BR-3 might recapitulate these distinct features and indicate that ROR2 is not per se associated with genomic instability.

In line with the rather mesenchymal, highly motile phenotype typically observed in basal-like cancer cells, ROR2 overexpression resulted in apparent defects in cell-cell-contacts with large gaps in confluent cell layers and membrane ruffles at cellular junctions (Fig. 1h). In particular, the distribution of the scaffolding protein ZO-1, which is required for the assembly of tight junctions, was severely disrupted in MCF-7, SK-BR-3, as well as BT-474 pROR2 cells. While ZO-1 was equally distributed at the plasma membrane in control cells, it clustered in large dots in pROR2 cells (Fig. 1i). A closer imaging of the cells by electron microscopy confirmed severe structural defects in cellular tight junctions, which were almost completely absent (Fig. 1j).

In its C-terminus ROR2 harbors a tyrosine kinase domain (TKD) as well as a proline-rich domain (PRD) that potentially mediate protein-protein interactions. In order to gain insight into the molecular mechanisms behind the aggressive function of ROR2, we transfected MCF-7 cells with *ROR2* constructs which either lacked the PRD (ΔPRD), or both PRD and TKD (ΔPRDΔTKD) (Fig. 1k+l, Fig. S2a + b). Using Boyden chamber assays we observed that only the double mutant showed a strong reduction in its invasive potential (Fig. 1m), suggesting that the TKD of ROR2 is essential for its invasion-promoting function.

### ROR2 enhances tumor invasion via RHO/ROCK signaling

**Fig. 2:**
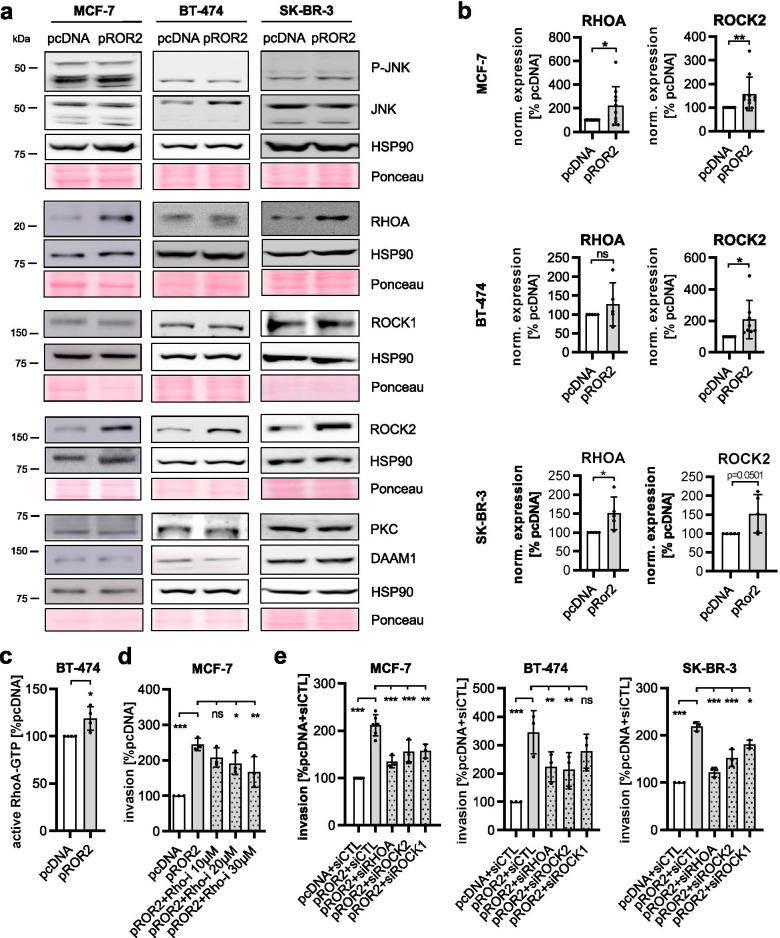
RHOA/ROCK mediate ROR2-induced tumor invasion. **a** pcDNA and pROR2 cells were characterized for the expression of non-canonical WNT signaling proteins by western blot. **b** Densitometric quantification of RHOA and ROCK2 in pcDNA and pROR2 cells normalized on HSP90 expression (mean ± SD, *p < 0.05, **p < 0.01, ns = not significant). **c** Active RHOA-GTP was measured in BT-474 cells by ELISA (mean ± SD, *n* = 4, *p < 0.05). **d** Invasion assay: MCF-7 pROR2 cells were treated for 96 h with the indicated concentration of Rhosin, a RHO inhibitor (mean ± SD, *n* = 3, *p < 0.05, **p < 0.01, ***p < 0.0001, ns = not significant). Significance was calculated with a one-way ANOVA with Dunnett’s multiple comparison test. **e** Cells were transfected with the indicated siRNAs (10 nM) and cell invasion was measured in Boyden chambers (mean ± SD, *n* = 3, *p < 0.05, **p < 0.01, ***p < 0.001, ns = not significant). Significance was calculated with a one-way ANOVA with Dunnett’s multiple comparison test

We had previously shown that overexpression of ROR2 does not affect the levels or localization of β-catenin in breast cancer cells and thus does not activate canonical WNT signaling [9]. To identify the oncogenic signaling responsible for the tumorigenic functions of ROR2, we characterized the ROR2-overexpressing cells by western blot for the expression of common non-canonical WNT signaling molecules (Fig. 2a). The most robust effect was a significant upregulation of the small GTPase Ras homolog family member A (RHOA) and its effector RHO-associated coiled-coil containing protein kinase 2 (ROCK2) in pROR2 cells (Fig. 2b, Fig. S2c). Since the increase in RHOA levels in BT-474 pROR2 cells was rather mild and did not reach statistical significance, we additionally measured the active GTP-bound form of RHOA by ELISA in this cell line (Fig. 2c). The result indicated that even though there were no major changes in total RHOA expression, RHOA-GTP levels were significantly increased in BT-474 overexpressing ROR2. Taken together, these results suggested that ROR2 activates WNT/PCP signaling.

To investigate whether the increased expression of RHOA is involved in the invasion-promoting role of ROR2, we treated MCF-7 pROR2 cells with the RHO inhibitor Rhosin, which blocks the activity of RHOA by inhibiting its interaction with its guanine nucleotide exchange factors (GEFs). As expected, this reduced the pro-invasive function of ROR2 in Boyden chamber assays in a concentration-dependent manner (Fig. 2d). Likewise, the same effect was seen after specific knockdown of RHOA, ROCK1 and ROCK2 by siRNA in all three ROR2-overexpressing breast cancer cell lines (Fig. 2e). In summary, the effect for the knockdown of ROCK1 was weaker than for ROCK2 and did not reach statistical significance in BT-474 cells. This suggested that even though the expression of ROCK1 had initially not been found to differ between cells transfected with empty vector or pROR2, the ROR2-induced increase in cancer cell invasiveness involved both, ROCK1 and ROCK2.

### ROR2 triggers expression of its putative non-canonical WNT ligands

**Fig. 3:**
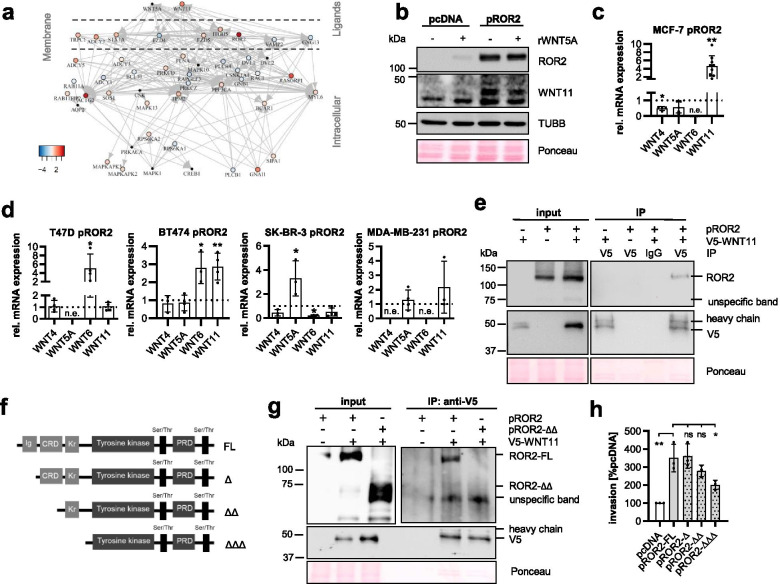
WNT11 is a novel ligand for ROR2 in humans. **a** RNA-Seq of MCF-7 pROR2 cells: Network of differentially expressed genes associated with non-canonical WNT signaling grouped according to their cellular localization. **b** MCF-7 cells were stimulated for 24 h with rWNT5A (100 ng/ml) and WNT11 expression was analyzed by western Blot. **c**+**d** Expression of the non-canonical WNT ligands was measured by qRT-PCR in MCF-7 (**c**) or the indicated ROR2-overexpressing human breast cancer cell lines (**d**) (mean ± SD, *n* = 3–9, *p < 0.05, *p* < 0.01, n.e. = not expressed). Expression values were calculated relative to the empty vector control cells. **e** Co-immunoprecipitation (Co-IP) of V5-WNT11 in MCF-7 pROR2 cells detects ROR2 by western blot. **f** Schematic representation of the ROR2 N-terminal deletion constructs. **g** Co-IP of V5-Wnt11 in MCF-7 expressing either pROR2-FL or pROR2-ΔΔ. **h** Cell invasion assays of MCF-7 expressing N-terminal ROR2 deletion constructs (mean ± SD, *n* = 3, *p < 0.01, **p = 0.0001, ns = not significant). Significance was calculated with a one-way ANOVA with Dunnett’s multiple comparison test

Next, we searched for potential non-canonical WNT ligands that could activate ROR2-induced WNT/PCP signaling. A comparison of MCF-7 empty vector and ROR2-overexpressing cells by RNA-Seq had previously identified 2860 differentially expressed genes (DEGs) in both cell lines [10]. To narrow down our search, we specifically filtered that list for genes that are part of the recently constructed network representing non-canonical WNT signaling [31]. Our analyses revealed the non-canonical WNT ligand *WNT11* as one of the most highly upregulated genes in the ROR2-overexpressing cells (Fig. 3a). The *WNT11* upregulation was confirmed at the protein level and was independent of WNT5A stimulation, the established ligand of human ROR2 (Fig. 3b). An analysis of the expression of other non-canonical WNT genes in MCF-7 cells revealed that there was no upregulation of any other typical non-canonical WNT ligands including *WNT4*, *WNT5A* or *WNT6* (Fig. 3c). To test whether the induction of WNT11 is specific for MCF-7 cells or a general phenomenon in breast cancer, we overexpressed ROR2 in different human breast cancer cell lines. Indeed, a comparable increase in *WNT11* levels was confirmed for BT-474 cells overexpressing ROR2 (Fig. 3d). The same trend was also observed in MDA-MB-231 cells, although it did not reach statistical significance. Other breast cancer cells reacted with the induction of different non-canonical WNT ligands, such as *WNT5A* in SK-BR-3 or *WNT6* in T-47D cells. Together, this supports the assumption that ROR2, when overexpressed, upregulates its own ligands in a cell context-dependent manner.

### WNT11 is a novel ligand for human ROR2

Based on these results, we hypothesized that WNT11 might act as a ligand for ROR2. WNT11 has already been identified as a ligand for ROR2 in zebrafish gastrulation [46]. However, it remained unknown whether WNT11 can also interact with ROR2 in humans and whether it might be responsible for its tumor-supporting function. To demonstrate that WNT11 indeed binds to human ROR2, we transiently transfected MCF-7 ROR2-overexpressing cells with a functionally active V5-tagged WNT11 (Fig. S2d). ROR2 was co-immunoprecipitated with V5-WNT11 (Fig. 3e), thus confirming their interaction.

ROR2 harbors three extracellular domains: an immunoglobulin-like (Ig-like), a cysteine-rich (CRD) as well as a Kringle domain. In Xenopus it has been shown that WNT proteins preferentially interact with the extracellular region of ROR2, in particular the CRD [47], while the Kringle domain seems to be important for receptor heterodimerization [48]. To investigate which domain is required for the pro-invasive function of ROR2, we cloned serial deletion constructs of ROR2 lacking the three extracellular domains and overexpressed them in MCF-7 cells (Fig. 3f). The deletion did not interfere with the localization of ROR2 which was consistently expressed at the plasma membrane in all transfected cells (Fig. S2e). Deletion of the first two domains abolished the interaction of V5-WNT11 and ROR2 in co-immunoprecipitations (Fig. 3g). While the lack of the first, Ig-like extracellular domain, did not affect the invasion-promoting effect of ROR2, a trend (*p* = 0.08) for reduced cancer cell invasiveness was observed when overexpressing ROR2 lacking the CRD (Fig. 3h). This effect was even more pronounced upon deletion of the Kringle domain. In summary, these observations indicated that WNT11 is a ligand for human ROR2 interacting with its CRD and that signal transduction involves receptor heterodimerization.

### WNT11 is responsible for the tumorigenic functions of ROR2 in breast cancer cells

**Fig. 4:**
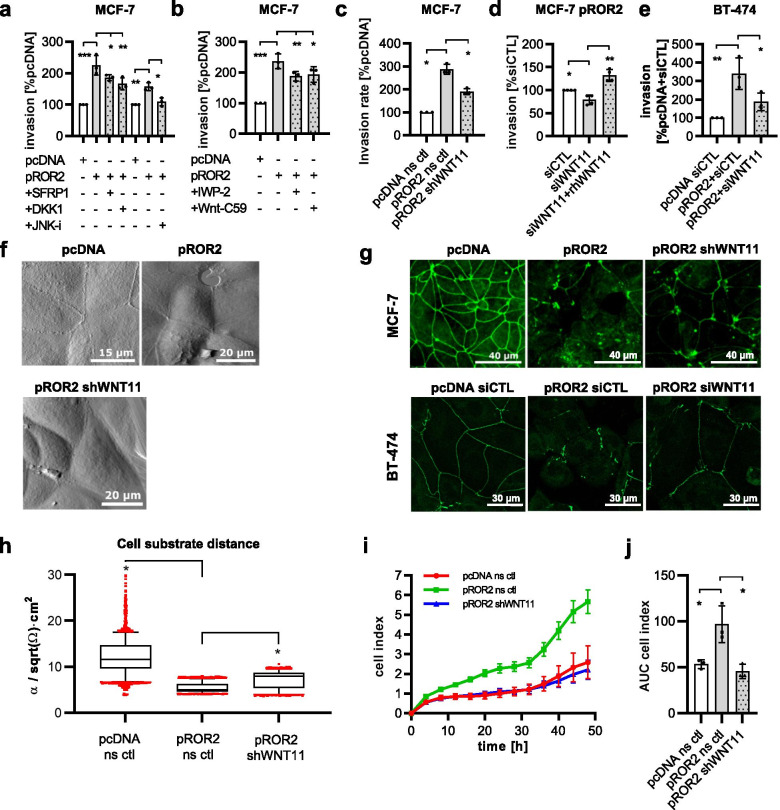
WNT11 mediates the pro-tumoral effects of ROR2. **a**+**b** Invasion assay: MCF-7 pcDNA or pROR2 cells were treated with WNT inhibitors (**a**) or Porcupine inhibitors (**b**) (mean ± SD, *n* = 3, *p < 0.05, **p < 0.01, ***p < 0.0001). Significance was calculated with a one-way ANOVA with Dunnett’s multiple comparison test. **c** Invasion assay of MCF-7 pROR2 cells stably expressing a non-sense control (ns ctl) or WNT11 (shWNT11) shRNA (mean ± SD, *n* = 3, *p < 0.001). Significance was calculated with a one-way ANOVA with Dunnett’s multiple comparison test. **d** Invasion of MCF-7 pROR2 cells transfected with control (siCTL) or WNT11 siRNA (siWNT11) +/− rhWNT11 (100 ng/ml) was assessed in Boyden chambers (mean ± SD, *n* = 3, *p < 0.05, **p < 0.001). Significance was calculated with a one-way ANOVA with Dunnett’s multiple comparison test. **e** Invasion assay of BT-474 pcDNA and pROR2 cells transfected with either siCTL or siWNT11 (mean ± SD, *n* = 3, *p < 0.05, **p < 0.001). Significance was calculated with a one-way ANOVA with Dunnett’s multiple comparison test. **f** AFM of cell-cell-junctions in the indicated cell lines. **g** Immunofluorescence for the tight junction protein ZO-1. **h** ECIS measurements of MCF-7 cells (box: 25-75th percentile, line at median, *p < 0.0001). Significance was calculated with a one-way ANOVA with Dunnett’s multiple comparison test. **i**+**j** xCELLigence measurements of MCF-7 cells (mean ± SD). Shown is one representative example (**i**) and a corresponding Area Under the Curve (AUC) analysis for all three independent experiments (mean ± SD, *p < 0.01). Significance was calculated with a one-way ANOVA with Dunnett’s multiple comparison test

We then aimed at further elucidating the consequences of the WNT11/ROR2 interaction for tumor cell function. Treatment of ROR2-overexpressing cells with soluble WNT ligand antagonists such as sFRP1 or DKK1 as well as an inhibitor blocking non-canonical WNT-JNK signaling inhibited ROR2-induced cancer cell invasiveness (Fig. 4a). In line with this, the two porcupine inhibitors IWP-2 and WNT-C59, which block the secretion of WNT ligands, had the same effect (Fig. 4b), underlining that a cell-intrinsic WNT ligand seemed to be responsible for the pro-invasive effect of ROR2. To confirm the involvement of WNT11 in this process, we generated MCF-7 pROR2 cells with a stable, shRNA-mediated knockdown of WNT11 (Fig. S3a). The invasiveness of these cells was significantly impaired (Fig. 4c). Similarly, a transient reduction of WNT11 expression by siRNA (Fig. S3b) resulted in decreased tumor cell invasion in MCF-7 and BT-474 pROR2 cells (Fig. 4d+e), an effect that was rescued by addition of recombinant WNT11 (Fig. 4d), thus confirming that WNT11 mediates a major part of the invasion-promoting effect of ROR2.

When the growth pattern and shape of MCF-7 pROR2 cells with stable WNT11 knockdown were analyzed, the cells resembled the empty vector control cells with only few membrane ruffles or gaps in the confluent cell layer (Fig. 4f). This amelioration of the defects in cell-cell-contacts was also mirrored in the distribution of ZO-1, which started to line again the cell-cell borders upon WNT11 depletion comparable to the empty vector control in MCF-7 and BT-474 cells (Fig. 4g). To evaluate the significance of these changes, we performed Electric Cell-Substrate Impedance Sensing (ECIS) measurements that permit the evaluation of the behavior of adherent cells, and are influenced by cellular morphology, adhesion and proliferation. Using this method, we were able to demonstrate that the aggressive phenotype of the pROR2 WNT11 knockdown cells showed a reversion towards the control cells, including a significantly higher cell substrate distance as well as a lower cell index compared to the ROR2-overexpressing cells (Fig. 4h-j).

### WNT11 mediates ROR2 signaling

**Fig. 5:**
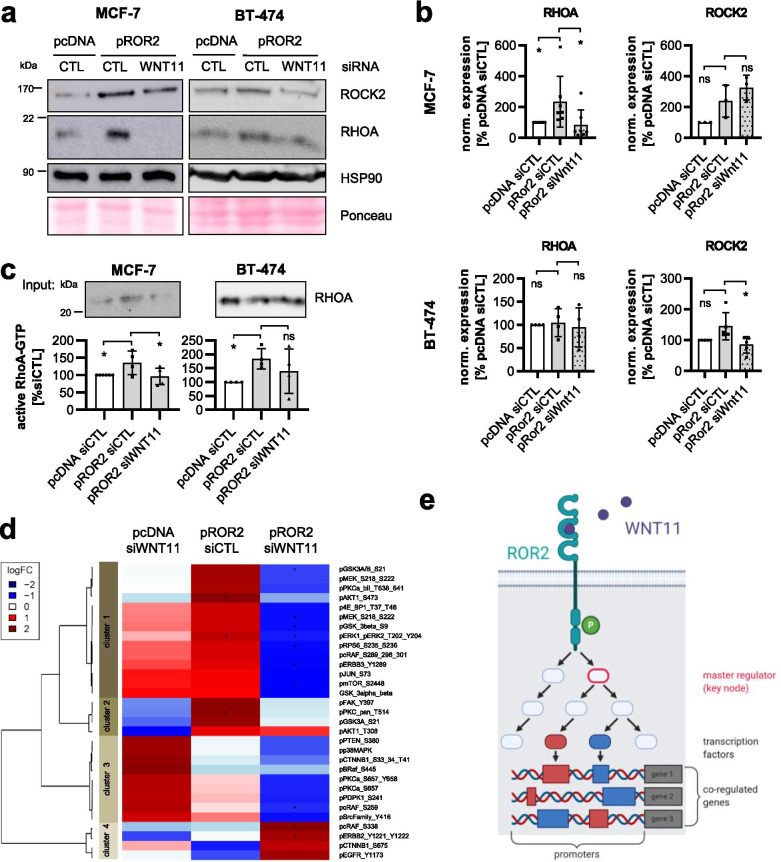
WNT11 mediates ROR2 signaling. **a**+**b** Western blot: RHOA and ROCK2 in MCF-7 and BT-474 pROR2 cells treated with control siRNA (siCTL) or siRNA against WNT11 (siWNT11) (**a**) with corresponding densitometric quantification normalized on HSP90 expression (**b**) (mean ± SD, *p < 0.05, ns = not significant). Significance was calculated with a one-way ANOVA with Dunnett’s multiple comparison test. **c** Levels of active RHOA-GTP in the indicated cells were assessed by ELISA (mean ± SD, *p < 0.05, ns = not significant). Significance was calculated with a one-way ANOVA with Dunnett’s multiple comparison test. **c** MCF-7 pcDNA and pROR2 siCTL/siWNT11 cells were characterized by RPPA for phospho-proteins associated with the WNT signaling pathway (*n* = 3, *p < 0.05). **d** Schematic representation of the master regulator analysis of ROR2/WNT11 signaling. The illustration was created with BioRender.com

Since we had identified RHOA and ROCK2 as critical signaling mediators for ROR2-induced invasion, we were interested in whether their activation was dependent on WNT11. Indeed, siRNA-mediated knockdown of WNT11 in ROR2-overexpressing MCF-7 cells antagonized the observed increase in RHOA, although no significant effect on ROCK2 expression was detectable (Fig. 5a+b). In contrast, in BT-474 pROR2 cells treated with WNT11 siRNA, the ROR2-induced increase in ROCK2 levels was abolished, while total RHOA levels remained unchanged. This is not surprising, as the BT-474 cells showed only an induction of active RHOA-GTP, but not total RHOA (Fig. 2a-c). Using an ELISA for the active, GTP-bound form of RHOA, we confirmed that ROR2 indeed triggered the activation of RHOA, an effect that was effectively counteracted by knockdown of WNT11 in both cell lines (Fig. 5c). Taken together, the results show that WNT11 is responsible for inducing active RHOA-GTP in ROR2-overexpressing cells, which can be accompanied by a concordant upregulation of total RHOA levels in a cell context-dependent manner. Activation of RHOA is linked to higher ROCK2 levels in MCF-7 and BT-474 cells, although this effect seems to be dependent on WNT11 only in the latter.

To gain further insight into additional pathways regulated by ROR2/WNT11, we characterized ROR2-overexpressing MCF-7 cells with siRNA-mediated WNT11 knockdown by RNA-Seq as well as RPPA. The RPPA chip contained antibodies against phospho-proteins involved in WNT signaling and associated pathways. Expression levels are presented in a heatmap in Fig. 5d. The array results showed that in particular proteins in cluster 1 and 2 were highly upregulated in pROR2 cells compared to empty vector control cells, and that these expression changes were reverted upon knockdown of WNT11, indicating that these sets of proteins were regulated through ROR2/WNT11.

We then aimed at identifying common master regulators that could mediate the activation of sets of these ROR2/WNT11 targets (Fig. 5e). In order to do so, we first identified differentially expressed genes (DEGs) between empty vector control and pROR2 cells based on the RNA-Seq results. Next, the promoters of DEGs were analyzed for enriched transcription factor binding sites to define sets of relevant transcription factors and relate them to upstream signal transduction pathways based on the regulatory pathway database TRANSPATH. The search for upstream signaling molecules that are responsible for regulating these sets of transcription factors finally identified the responsible master regulators. The master regulator analysis of empty vector and pROR2 cells revealed *PIK3CA and RHOA* as master regulators of signaling in ROR2-overexpressing cells (Table S4, Fig. S4). This fits to the results of the RPPA characterization with several signaling molecules of the PI3K pathway present in the WNT11-regulated clusters 1 and 2 (e.g. P-AKT, P-mTOR, P-RPS6) (Fig. 5d). Moreover, it suggests that WNT11 is able to activate PI3K signaling, indicating that it might be responsible for the activation of PI3K in pROR2 cells. In line with this hypothesis, *PIK3CA* was no longer detectable as a master regulator in pROR2 cells after knockdown of WNT11 (Table S5). Taken together, these results imply that WNT11 is able to activate tumor-promoting signaling pathways in breast cancer cells via its interaction with ROR2.

### WNT11/ROR2 are highly expressed in breast cancer brain metastases and are associated with poor patient survival

**Fig. 6:**
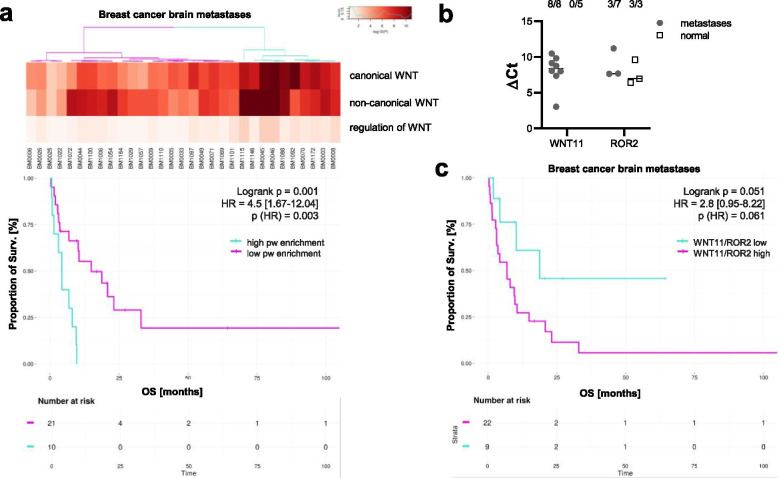
ROR2/WNT11 are expressed in metastatic breast cancer and associated with poor survival. **a** Pathway (pw) enrichment for RNA-Seq data from 31 patients with brain metastases given for different WNT subpathways. Significance was calculated with a log rank test. **b** qRT-PCR: Expression of *ROR2* and *WNT11* in samples of human brain metastases (line at median). The upper numbers indicate the number of samples with positive signals out of all investigated samples. **c** Kaplan-Meier survival curves showing the OS of metastatic patients based on their averaged *WNT11* and *ROR2* expression. The separation high/low was computed based on an optimal cutoff using the maxstat method. Significance was calculated with a log rank test

While studies have suggested hyperactive WNT signaling in primary breast cancers, it is still not clear whether the same holds true for metastases which are known to differ from the primary tumor [7]. We therefore collected samples from 31 patients with brain metastases and characterized them by RNA-Seq. With the obtained data, we performed a pathway enrichment analysis looking at three different gene sets, either associated with canonical, non-canonical, or regulation of WNT signaling [31] (Fig. 6a). Interestingly, genes associated with non-canonical WNT signaling were highly enriched in almost all samples, and the enrichment was more pronounced than for canonical WNT signaling. The cluster of patients with the highest enrichment was characterized by significantly shorter overall survival.

Next, we isolated RNA from seven metastases as well as normal brain tissue and analyzed the expression of *ROR2* and its ligand *WNT11* by quantitative real-time PCR (Fig. 6b). While *ROR2* was present in half of the metastases and all control samples, *WNT11* was undetectable in normal brain tissue, but highly expressed in all metastases, thus further pointing towards its important role in metastatic growth. To confirm the role of *ROR2/WNT11* in metastasis, we correlated the expression levels of *ROR2* and *WNT11* in the metastatic tissue with patient outcome using the gene expression data obtained by RNA-Seq. Indeed, patients with high expression of *ROR2* plus *WNT11* had the shortest overall survival (Fig. 6c). Although the results did not quite reach significance due to the limited number of patient samples, the results do indicate that WNT11/ROR2 exerts an unfavorable role in brain metastasis of breast cancer patients in vivo.

## Discussion

Previously, we had described a gene expression signature comprising 76 genes regulated by the WNT co-receptor ROR2 which grouped primary breast cancer patients into two clusters with significant differences in MFS [10]. Moreover, we had detected high expression levels of *ROR2* in breast cancer brain metastases [8], suggesting its active involvement in tumor progression. Now, we explored the molecular mechanisms underlying these observations and showed that ROR2 confers an aggressive phenotype to breast cancer cells that is linked to basal-like features and a high invasive potential. We identified WNT11 as a ligand for human ROR2 that activates WNT/PCP signaling and is responsible for the induction of tumor invasion. *WNT11* is highly expressed in brain metastases and is associated with poor patient survival when co-expressed with *ROR2*. Some ROR2-positive breast cancers can be characterized by a BRCAness-like gene expression signature in a cell context-dependent manner with an enhanced susceptibility to PARP inhibition, thereby opening new possibilities for targeted treatment.

ROR2 has been reported to act either as a tumor suppressor or an oncogene depending on the type of tumor [15]. This is not surprising since WNT signaling responses are highly cell context-dependent and involve a multitude of WNT subnetworks that result in diverse functional outcomes. WNT ligands have been shown to compete for receptor binding on the cell surface [49]. Hence, the combination of available ligand, receptor and co-receptor seems to dictate which subnetworks are activated. Our own results indicate that in breast cancer especially non-canonical WNT signaling is highly active [8–10]. Correspondingly, a negative correlation between ROR2 and active canonical WNT signaling was revealed in human breast tumors based on the TCGA database [14]. So far, available evidence indicates that ROR2 acts as an oncogene in breast cancer. In vitro studies have revealed a stimulatory function of ROR2 on breast cancer cell invasion and migration, mostly through induction of an EMT-like cancer cell phenotype [9, 12, 13]. Moreover, the treatment of MCF-7 cells with increasing doses of tamoxifen generated resistant clones that exhibited an EMT-like phenotype and were characterized by elevated ROR2 levels [50]. This fits our detection of morphological alterations with a switch to the aggressive, highly motile basal-like subtype in ROR2-overexpressing cells. In mice, ROR2-positive tumors are associated with accelerated tumor growth and a shorter survival time, confirming the tumor-promoting role of ROR2 in vivo [14, 51]. Interestingly, overexpression of ROR2 induced a BRCAness-like gene expression profile in MCF-7 breast cancer cells. To our knowledge, ROR2 has not yet been associated with genomic instability, thus necessitating further research to understand this finding, in particular the identification of context-dependent factors that influence the development of ROR2-associated BRCAness traits in some cancer cells. However, the observation that certain ROR2-overexpressing cancers become susceptible to PARP inhibition paves the way for a new approach for treating such ROR2-positive tumors.

Although initial studies in Xenopus and *C. elegans* had identified WNT binding domains in ROR2 [47, 52], and a physical interaction of ROR2 with WNT11 had been described in zebrafish [46], WNT5A was still believed to be the sole ligand for ROR2 in humans. The newly identified interaction between human ROR2 and WNT11 suggests a similar promiscuity for ROR2 in ligand binding compared to the FZD receptors. The observation that ROR2 is able to upregulate the expression of several non-canonical WNT ligands in a cell line-specific context, highlights WNT6 as another potential ligand for ROR2. In contrast, WNT4 was not differentially expressed upon ROR2 overexpression in the investigated breast cancer cell lines. However, it cannot be excluded that it might act as a ROR2 ligand in a particular context.

It remains unclear whether WNT11 stimulation couples ROR2 to additional co-receptors (e.g. FZDs) as it has been observed for WNT5A [49]. Interestingly, while the deletion of the Ig and CRD domain of ROR2 decreased its invasion-promoting effect, an additional reduction was observed upon deletion of the Kringle domain. As the Kringle domain has been shown to contain a lysine-binding site that could potentially mediate receptor-receptor interactions [53], this observation might point to the involvement of other receptors in the WNT11/ROR2 complex. ROR2 is able to heterodimerize with PTK7 to stimulate vertebrate WNT/PCP signaling [54], raising the question whether apart from the FZDs it might also serve as candidate for a possible complex formation.

*WNT11* was reported to be highly amplified in 6.2% of breast cancer patients [11] and observed in specific tumor cell subpopulations [14]. Moreover, *WNT11* was recently identified as one of three WNT genes mutated specifically in breast cancer metastases compared with matched primary tumors [7]. While our data reveal a high expression of *WNT11* in brain metastases and poor patient survival when co-expressed with *ROR2*, the effect of WNT11 signaling might vary in different receptor contexts. In our cell line models WNT11/ROR2 signaling resulted in activation of RHOA/ROCK and thus elicited a typical WNT/PCP response. This is in line with studies of WNT11 signaling in zebrafish and mouse [14, 46]. The observation was further confirmed by our network analysis which identified *RHOA* as well as *PIK3CA* as master regulators of ROR2 signaling. Since *PIK3CA* was lost as a master regulator upon *WNT11* knockdown, this suggests that WNT11 might integrate WNT and PI3K/AKT signaling. Although this requires further proof, similar observations have been claimed for prostate cancer [55].

## Conclusions

Taken together, we show for the first time that ROR2 can trigger an autocrine, invasion-promoting signaling response via RHO/ROCK by inducing expression of its own ligand, WNT11, in human breast cancer. Obviously, the notion of WNT5A as the sole ligand for human ROR2 no longer holds true which opens novel insights in the regulation of non-canonical WNT signaling in cancer. ROR2/WNT11 upregulation is especially relevant in the metastatic situation since it confers poor prognosis on the one hand, but also sensitivity towards PARP inhibitors on the other, thus providing a promising therapeutic target worth further exploration.

## Supplementary Information

**Additional file 1: Fig. S1.** BRCAness signature in SK-BR-3 cells. **Fig. S2.** Modulation of the WNT11 and ROR2 expression in MCF-7 cells. **Fig. S3.** Knockdown of WNT11 in MCF-7 pROR2 cells. **Fig. S4.** PIK3CA and RHOA are master regulators of ROR2 signaling. **Table S1.** Antibodies used for RPPA. **Table S2.** Antibodies used for western blots. **Table S3.** Primers used for quantitative real-time PCR. **Table S4.** Master regulator analysis of MCF-7 pcDNA siWNT11 versus pROR2 siCTL cells. **Table S5.** Master regulator analysis of MCF-7 pcDNA siWNT11 versus pROR2 siWNT11 cells.

## Data Availability

The RNA-Seq data of breast cancer brain metastases and of MCF-7 and SK-BR-3 pcDNA/pROR2 cells with/without siRNA treatment have been uploaded to the GEO repository under the identifiers GSE74383, GSE161864 and GSE161865. All other data supporting the findings of this study are available within the article and the supplemental information, or from the corresponding author upon reasonable request.

## Abbreviations

AFM: Atomic force microscopy
CRD: Cysteine-rich domain
DEG: Differentially expressed gene
ECIS: Electric Cell-Substrate Impedance Sensing
ELISA: Enzyme-linked immunosorbent assay
EMT: Epithelial-to-mesenchymal transition
FCS: Fetal calf serum
FZD: Frizzled
GSVA: Gene Set Variation Analysis
HR: Hazard ratio
MFS: Metastasis-free survival
OS: Overall survival
PARP: Poly (ADP-ribose) polymerase
PCP: Planar cell polarity
PFS: Progression-free survival
qRT-PCR: Quantitative real-time PCR
ROR2: Receptor tyrosine kinase-like orphan receptor 2
RPPA: Reverse phase protein array
RSEM: RNA-Seq by expectation-maximization
SD: Standard deviation
TCGA: The Cancer Genome Atlas
TKD: Tyrosine kinase domain
