# Supplementary material for: WNT11/ROR2 signaling is associated with tumor invasion and poor survival in breast cancer
Source: J Exp Clin Cancer Res. 2021 Dec 15;40:395. doi: 10.1186/s13046-021-02187-z (PMC8672621; doi:10.1186/s13046-021-02187-z)
Supplement: Supplementary file 1 — Additional file 1: Fig. S1. BRCAness signature in SK-BR-3 cells. Fig. S2. Modulation of the WNT11 and ROR2 expression in MCF-7 cells. Fig. S3. Knockdown of WNT11 in MCF-7 pROR2 cells. Fig. S4. PIK3CA and RHOA are master regulators of ROR2 signaling. Table S1. Antibodies used for RPPA. Table S2. Antibodies used for western blots. Table S3. Primers used for quantitative real-time PCR. Table S4. Master regulator analysis of MCF-7 pcDNA siWNT11 versus pROR2 siCTL cells. Table S5. Master regulator analysis of MCF-7 pcDNA siWNT11 versus pROR2 siWNT11 cells. [file 13046_2021_2187_MOESM1_ESM.docx]

Supplemental Information

Figure S1.


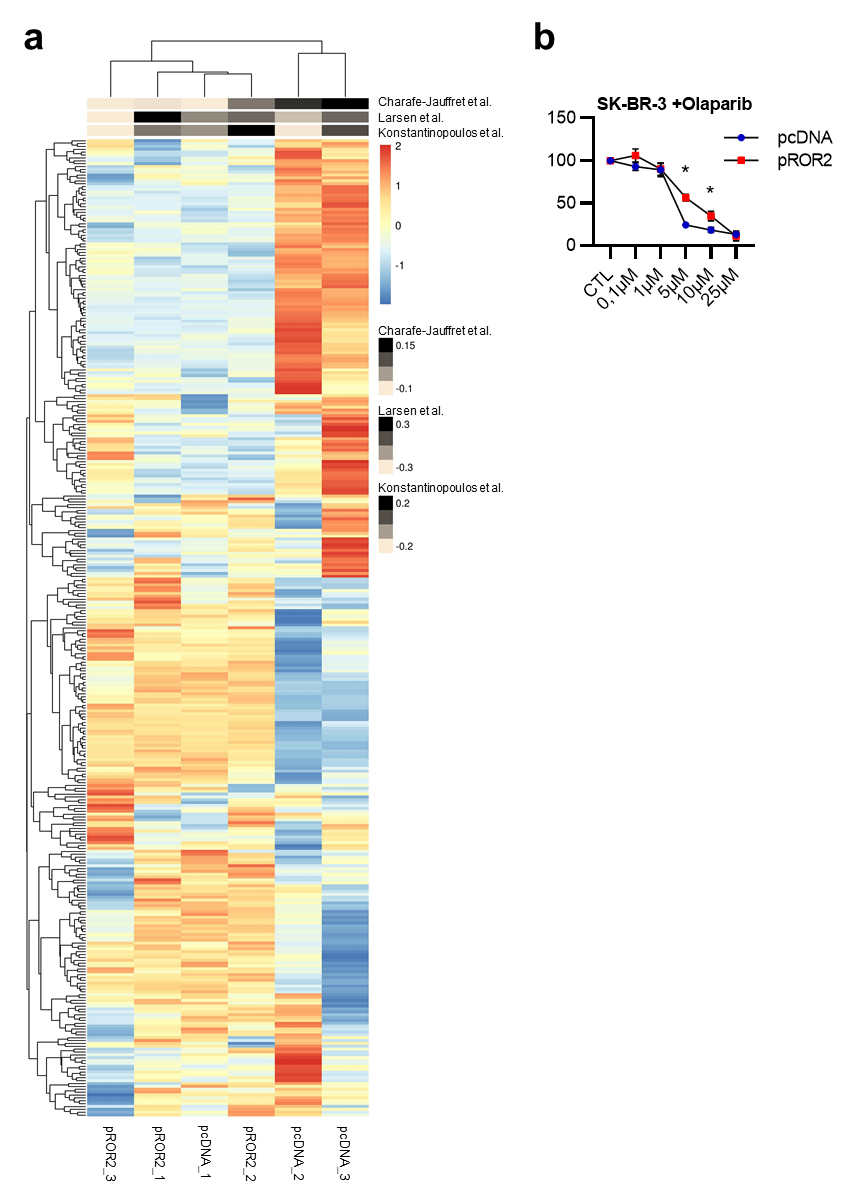


**Fig. S1: BRCAness signature in SK-BR-3 cells.** ***a,*** GSVA analysis for three independently published BRCAness gene expression signatures for SK-BR-3 cells overexpressing either an empty vector (pcDNA) or ROR2 overexpression plasmid (pROR2). Shown are three biological replicates each. ***b,*** MTT assay: SK-BR-3 pcDNA and pROR2 cells were treated for 96 h with the indicated concentrations of olaparib (mean±SD, n=3, *p<0.05).

Figure. S2.


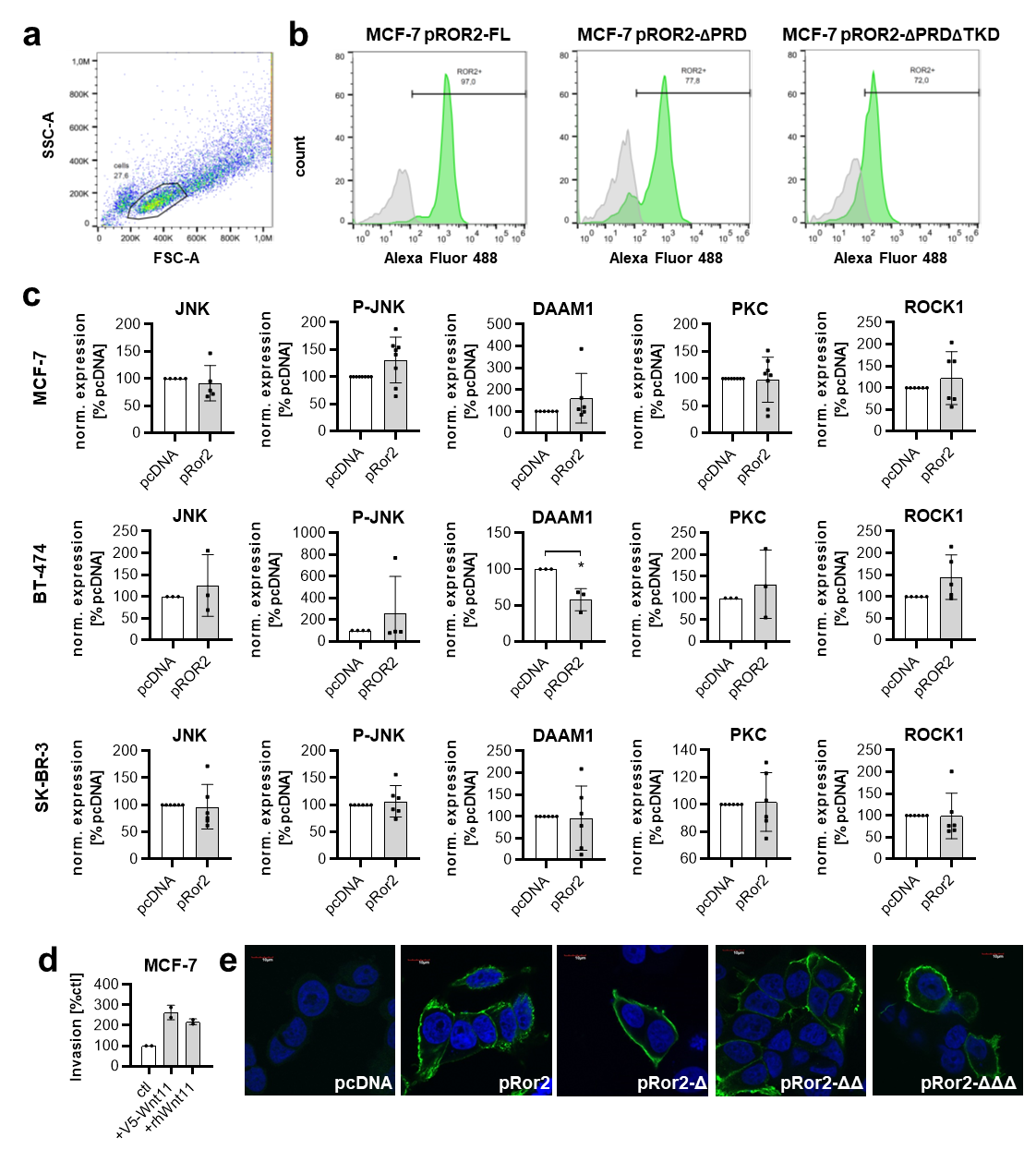


**Fig. S2: Modulation of the WNT11 and ROR2 expression in MCF-7 cells. *a+b,*** MCF-7 cells stably transfected with either ROR2 full-length (pROR2-FL) or C-terminal deletion constructs were analyzed by flow cytometry for their ROR2 expression. Cell populations were gated based on FSC vs SSC plots (a) and representative histograms for the expression of ROR2 (green) in comparison to the isotype control (grey) in the gated cell population are shown (b). ***c,*** Western Blot: Densitometric quantification of the indicated proteins in MCF-7, BT-474 and SK-BR-3 cells overexpressing either empty vector (pcDNA) or human ROR2 (pROR2). Protein expression was normalized on the housekeeping protein (mean±SD, *p<0.01). All significant values were marked with an asterisk. ***d,*** Invasion assay of MCF-7 cells transiently transfected with V5-tagged WNT11 (mean±SD, n=2). ***e,*** Confocal microscopy: Immunofluorescence staining of MCF-7 cells transfected with the serial N-terminal ROR2 deletion constructs (blue: DAPI, green: ROR2). Scale bar: 10 µm

Figure. S3.

**a**

**b**

**Fig. S3: Knockdown of WNT11 in MCF-7 pROR2 cells. *a***, qRT-PCR: *WNT11* knockdown was confirmed in MCF-7 cells stably overexpressing either a non-sense control (ns ctl) or a shRNA directed against *WNT11* (mean±SD, n=3, *p<0.05). Significance was calculated with a one-way ANOVA with Dunnett‘s multiple comparison test. ***b,*** *WNT11* expression was measured by qRT-PCR in MCF-7 pROR2 cells transiently transfected with a control siRNA (siCTL) or siRNA against WNT11 (siWNT11) (mean±SD, n=4, *p<0.001).

Figure. S4.


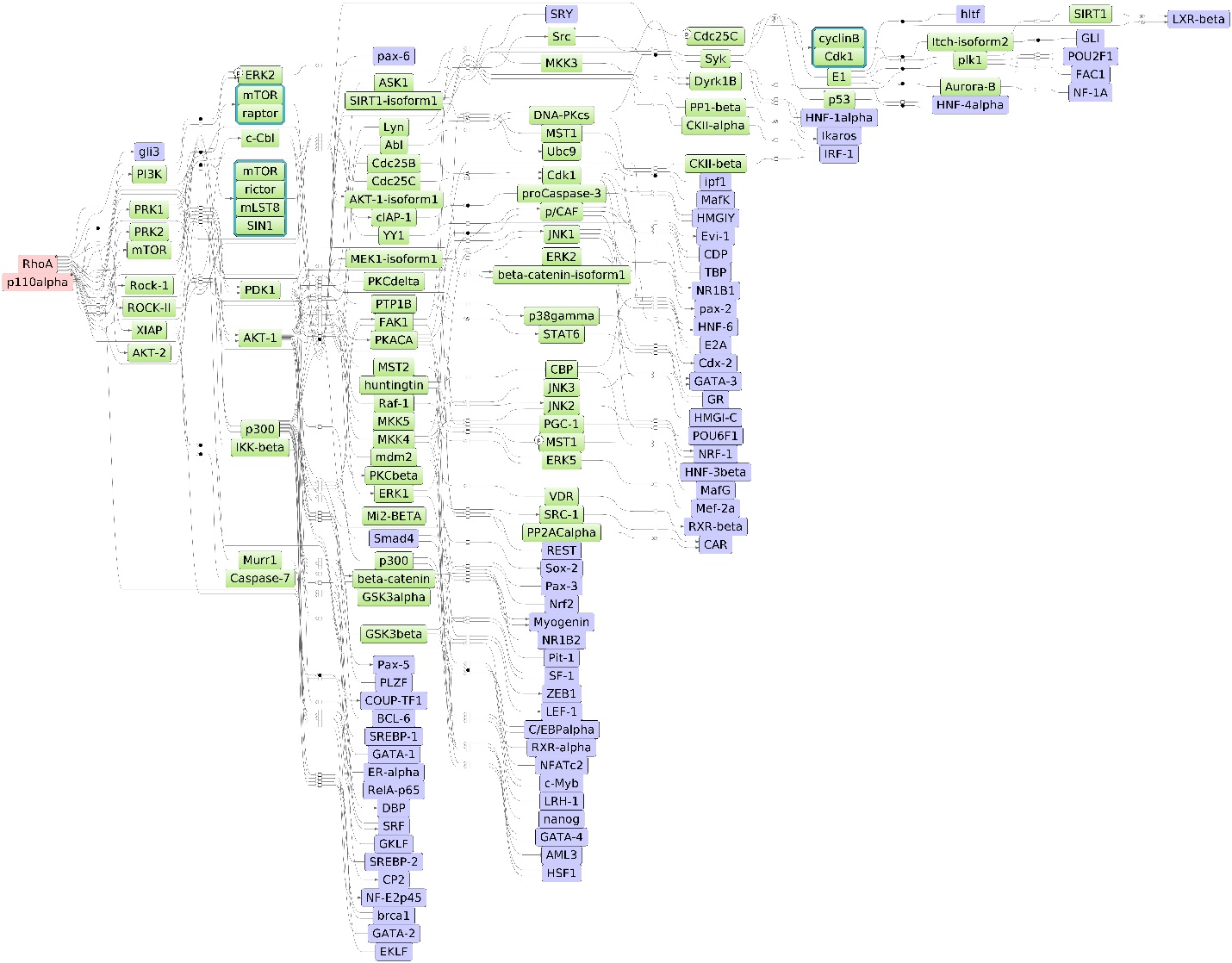


**Fig. S4: PIK3CA and RHOA are master regulators of ROR2 signaling.** Master regulatory network based on RNA-Seq data of MCF-7 pcDNA vs pROR2 cells. Red: master regulators, purple: regulated transcription factors, green; connecting molecules.

Table S1.

Antibodies used for RPPA.

Table S2.

Antibodies used for western blots.

| **Gene.Symbol** | **Supplier** | **Antibody.ID** | **Host** |
| --- | --- | --- | --- |
| DAAM1 | santa cruz | sc-100942 | mouse |
| GAPDH | santa cruz | sc-32233 | mouse |
| HSP90 | santa cruz | sc-13119 | mouse |
| JNK | CST | 9252 | rabbit |
| P-JNK | santa cruz | sc-6254 | mouse |
| PKC | santa cruz | sc-10800 | mouse |
| RHOA | santa cruz | sc-418 | mouse |
| ROCK1 | santa cruz | sc-17794 | mouse |
| ROCK2 | santa cruz | sc-398519 | mouse |
| ROR2 | santa cruz | sc-98486 | rabbit |
| ROR2 | santa cruz | sc-80329 | mouse |
| TUBA | Millipore | 05-829 | mouse |
| V5-Tag | CST | 13202 | rabbit |
| WNT11 | abcam | ab31962 | rabbit |

Table S3.

Primers used for quantitative real-time PCR.

| **primer** | **sequence forward primer (5'-3')** | **sequence reverse primer (5'-3')** |
| --- | --- | --- |
| hsWNT4_fw/rv150 | CTGAAGGAGAAGTTTGATGGTG | TGTCCTGCTCACAGAAGTC |
| hsWNT5A_fw/rv109 | AGGGCTCCTACGAGAGTGCT | GACACCCCATGGCACTTG |
| hsWNT6_fw/rv150 | GGAGCGTTTAAAGGACACTG | GATACTAACCTCACCCACCA |
| hsWNT11_fw/rv102 | CTCGGAACTCGTCTATCTG | GTTGGATGTCTTGTTGCAC |
| hsROR2_fw/rv143 | TTCTTCTTGGTTTGCATGTG | CTGATCTCTTTGAGTTTGGC |
| hsHPRT1_fw/rv89 | TATGCTGAGGATTTGGAAAGG | CATCTCCTTCATCACATCTCG |
| hsGNB2L1_fw/rv84 | AACCCTATCATCGTCTCCT | CAATGTGGTTGGTCTTCAG |

Table S4.

Master regulator analysis of MCF-7 pcDNA siWNT11 versus pROR2 siCTL cells.

| **Gene Symbol** | **Master molecule name** | **FDR** |
| --- | --- | --- |
| RHOA | RhoA(h) | 0,002 |
| RAD23A | Rad23A(h) | 0,002 |
| CUX1 | CDP(h) | 0,002 |
| UBE2K | HIP2(h) | 0,002 |
| AMER1 | FAM123B(h) | 0,003 |
| CREBBP | CBP(h) | 0,004 |
| NR3C1 | GR(h) | 0,005 |
| CEBPA | C/EBPalpha(h) | 0,005 |
| CHUK | IKK-alpha:IKK-beta{p}:(IKK-gamma)2 | 0,006 |
| IKBKB | IKK-alpha:IKK-beta{p}:(IKK-gamma)2 | 0,006 |
| IKBKG | IKK-alpha:IKK-beta{p}:(IKK-gamma)2 | 0,006 |
| RARA | NR1B1(h) | 0,006 |
| PIK3CA | p110alpha(h) | 0,007 |
| PKN2 | PRK2(h) | 0,009 |
| CASP6 | proCaspase-6(h) | 0,01 |
| PRSS1 | trypsin-1(h) | 0,01 |
| EP300 | p300(h) | 0,011 |
| NLRC4 | CLAN(h) | 0,011 |
| INS | insulin(h) | 0,011 |
| PHLPP1 | PHLPP(h) | 0,012 |
| PHLPP2 | phlpp2(h) | 0,012 |
| PIK3CD | p110delta(h) | 0,013 |
| MECOM | Evi-1(h) | 0,013 |
| RARB | NR1B2(h) | 0,013 |
| CASP1 | Caspase-1(h) | 0,014 |
| CAMK2A | CamKII(h) | 0,015 |
| CAMK2B | CamKII(h) | 0,015 |
| CAMK2D | CamKII(h) | 0,015 |
| CAMK2G | CamKII(h) | 0,015 |
| RAC3 | Rac3(h) | 0,015 |
| RHOG | RhoG(h) | 0,015 |
| REST | REST(h) | 0,015 |
| HNF4A | HNF-4alpha(h) | 0,015 |
| RAC2 | Rac2(h) | 0,016 |
| NEK2 | Nek2A(h) | 0,017 |
| TRIM32 | HT2A(h) | 0,017 |
| TGFBR2 | TGFbetaR-II(h) | 0,018 |
| GATA3 | GATA-3(h) | 0,019 |
| HSF1 | HSF1(h) | 0,019 |
| NANOG | nanog(h) | 0,02 |
| HTT | huntingtin(h) | 0,021 |
| ROCK2 | ROCK-II(h) | 0,022 |
| FURIN | PACE(h) | 0,022 |
| ESR1 | ER-alpha(h) | 0,022 |
| PDPK1 | PDK1-isoform2(h) | 0,023 |
| EGF | EGF:ErbB1{pY}:ErbB2{pY}:Src | 0,023 |
| EGFR | EGF:ErbB1{pY}:ErbB2{pY}:Src | 0,023 |
| ERBB2 | EGF:ErbB1{pY}:ErbB2{pY}:Src | 0,023 |
| SRC | EGF:ErbB1{pY}:ErbB2{pY}:Src | 0,023 |
| NFATC2 | NFATc2(h) | 0,025 |
| MYB | c-Myb(h) | 0,025 |
| CDC42 | Cdc42-isoform2(h) | 0,026 |
| PPP1CB | PP1-beta(h) | 0,027 |
| CDK1 | Cdk1(h) | 0,027 |
| CASP7 | proCaspase-7(h) | 0,029 |
| RELA | RelA-p65(h) | 0,029 |
| PRKCD | PKCdelta(h){pT507}{pS645}{pS664} | 0,03 |
| IGF1R | IGF-1R(h) | 0,03 |
| CASP2 | Caspase-2(h) | 0,031 |
| HMGA1 | HMGIY(h) | 0,031 |
| PTK2B | Pyk2-isoform1(h) | 0,032 |
| FRAT1 | Frat1(h) | 0,032 |
| PRKCG | PKCgamma(h) | 0,033 |
| SUMO1 | sumo1(h) | 0,033 |
| AKT1 | AKT(h){ub}n | 0,034 |
| AKT2 | AKT(h){ub}n | 0,034 |
| PRKCI | PKCiota(h) | 0,036 |
| DRD1 | D1(h) | 0,036 |
| PDK1 | pyruvate dehydrogenase (lipoamide) kinase isozyme 1, mitochondrial(h) | 0,037 |
| BAX | Bax(h) | 0,038 |
| CASP10 | Caspase-10(h) | 0,04 |
| SENP5 | Senp5-isoform1(h) | 0,04 |
| DLG4 | PSD-95(h) | 0,04 |
| DUSP22 | JKAP(h) | 0,042 |
| MTOR | mTOR(h):rictor(h) | 0,042 |
| RICTOR | mTOR(h):rictor(h) | 0,042 |
| EPHB2 | EPHB2(h) | 0,042 |
| SGK1 | SGK-1(h){pT256} | 0,042 |
| FAS | Fas(h) | 0,044 |
| BRCA1 | brca1(h) | 0,045 |
| SAE1 | Aos1(h):SAE2-isoform1(h) | 0,045 |
| UBA2 | Aos1(h):SAE2-isoform1(h) | 0,045 |
| PPP1R9B | neurabin-II(h) | 0,047 |
| MUL1 | MAPL(h) | 0,048 |
| SQSTM1 | p62(h) | 0,049 |

Table S5.

Master regulator analysis of MCF-7 pcDNA siWNT11 versus pROR2 siWNT11 cells.

| **Gene Symbol** | **Master molecule name** | **FDR** |
| --- | --- | --- |
| PRSS1 | trypsin-1(h) | 0,001 |
| RHOA | RhoA(h) | 0,002 |
| CUX1 | CDP(h) | 0,003 |
| IGF1R | IGF-1R(h) | 0,003 |
| EGF | EGF:ErbB1{pY}:ErbB2{pY}:Src | 0,008 |
| EGFR | EGF:ErbB1{pY}:ErbB2{pY}:Src | 0,008 |
| ERBB2 | EGF:ErbB1{pY}:ErbB2{pY}:Src | 0,008 |
| SRC | EGF:ErbB1{pY}:ErbB2{pY}:Src | 0,008 |
| YY1 | YY1(h) | 0,01 |
| ESR1 | ER-alpha(h) | 0,01 |
| DUSP22 | JKAP(h) | 0,011 |
| NR3C1 | GR(h) | 0,011 |
| CEBPA | C/EBPalpha(h) | 0,011 |
| PPP1CB | PP1-beta(h) | 0,012 |
| PPP1R9B | neurabin-II(h) | 0,012 |
| MYB | c-Myb(h) | 0,014 |
| MECOM | Evi-1(h) | 0,015 |
| PRKCD | PKCdelta(h),PKCdelta(h){pT507}{pS645}{pS664} | 0,016 |
| RARA | NR1B1(h) | 0,018 |
| GATA3 | GATA-3(h) | 0,02 |
| RARB | NR1B2(h) | 0,02 |
| CHUK | IKK-alpha:IKK-beta{p}:(IKK-gamma)2 | 0,021 |
| IKBKB | IKK-alpha:IKK-beta{p}:(IKK-gamma)2 | 0,021 |
| IKBKG | IKK-alpha:IKK-beta{p}:(IKK-gamma)2 | 0,021 |
| INS | insulin(h) | 0,021 |
| NANOG | nanog(h) | 0,021 |
| LMTK2 | KPI-2(h) | 0,022 |
| NFATC2 | NFATc2(h) | 0,022 |
| KRAS | K-Ras(h) | 0,023 |
| PTK2B | Pyk2-isoform1(h) | 0,023 |
| NEK2 | Nek2A(h),Nek2A(h){p} | 0,024 |
| CASP2 | proCaspase-2(h) | 0,024 |
| GABPA | GABP-alpha(h) | 0,024 |
| HSF1 | HSF1(h) | 0,024 |
| DRD1 | D1(h) | 0,026 |
| GABPB1 | GABP-beta(h) | 0,026 |
| RAD23A | Rad23A(h) | 0,027 |
| AMER1 | FAM123B(h) | 0,027 |
| HMGA1 | HMGIY(h) | 0,027 |
| PPP1CA | PP1-alpha(h),PP1-alpha1(h),PP1-alpha2(h) | 0,03 |
| RAC3 | Rac3(h) | 0,034 |
| RHOG | RhoG(h) | 0,034 |
| PPP1CC | PP1-gamma1(h) | 0,035 |
| ICMT | ICMT(h) | 0,035 |
| BAIAP2 | IRSp53(h) | 0,036 |
| IRS4 | IRS-4(h) | 0,037 |
| DUSP10 | MKP-5-isoform1(h) | 0,038 |
| ARHGDIA | RhoGDI-1(h) | 0,038 |
| HTT | huntingtin(h) | 0,039 |
| RAC2 | Rac2(h) | 0,039 |
| RELA | RelA-p65(h) | 0,042 |
| CDC42 | Cdc42-isoform1(h),Cdc42-isoform2(h) | 0,043 |
| FURIN | PACE(h) | 0,0435 |
| CAMK2A | CamKII(h) | 0,045 |
| CAMK2B | CamKII(h) | 0,045 |
| CAMK2D | CamKII(h) | 0,045 |
| CAMK2G | CamKII(h) | 0,045 |
| ROCK2 | ROCK-II(h) | 0,047 |
| FAS | Fas(h) | 0,047 |
